# Evaluation of a new software prototype for frameless radiosurgery of arteriovenous malformations

**DOI:** 10.1186/s13014-019-1422-x

**Published:** 2019-12-02

**Authors:** Daniel Schmidhalter, Dominik Henzen, Evelyn Herrmann, Werner Volken, Paul-Henry Mackeprang, Ekin Ermis, Hossein Hemmatazad, Jonas Honegger, Benjamin Haas, Michael K. Fix, Peter Manser

**Affiliations:** 1 Division of Medical Radiation Physics and Department of Radiation Oncology, Inselspital, Bern University Hospital, and University of Bern, Berne, Switzerland; 2 Varian Medical Systems Imaging Laboratory GmbH, CH-5405 Dättwil, Switzerland

**Keywords:** Arteriovenous malformation, Digital subtraction angiography, Radiosurgery, Frameless, Non-invasive, Brain Clinic

## Abstract

**Background:**

In order to locate an arteriovenous malformation, typically, a digital subtraction angiography (DSA) is carried out. To use the DSA for target definition an accurate image registration between CT and DSA is required. Carrying out a non-invasive, frameless procedure, registration of the 2D-DSA images with the CT is critical. A new software prototype is enabling this frameless procedure. The aim of this work was to evaluate the prototype in terms of targeting accuracy and reliability based on phantom measurements as well as with the aid of patient data. In addition, the user’s ability to recognize registration mismatches and quality was assessed.

**Methods:**

Targeting accuracy was measured with a simple cubic, as well as with an anthropomorphic head phantom. Clearly defined academic targets within the phantoms were contoured on the CT. These reference structures were compared with the structures generated within the prototype. A similar approach was used with patient data, where the clinically contoured target served as the reference structure.

An important error source decreasing the target accuracy comes from registration errors between CT and 2D-DSA. For that reason, the tools in BC provided to the user to check these registrations are very important. In order to check if the user is able to recognize registration errors, a set of different registration errors was introduced to the correctly registered CT and 2D-DSA image data sets of three different patients. Each of six different users rated the whole set of registrations within the prototype.

**Results:**

The target accuracy of the prototype was found to be below 0.04 cm for the cubic phantom and below 0.05 cm for the anthropomorphic head phantom. The mean target accuracy for the 15 patient cases was found to be below 0.3 cm.

In the registration verification part, almost all introduced registration errors above 1° or 0.1 cm were detected by the six users. Nevertheless, in order to quantify and categorize the possibility to detect mismatches in the registration process more data needs to be evaluated.

**Conclusion:**

Our study shows, that the prototype is a useful tool that has the potential to fill the gap towards a frameless procedure when treating AVMs with the aid of 2D-DSA images in radiosurgery. The target accuracy of the prototype is similar to other systems already established in clinical routine.

## Background

Arteriovenous malformations (AVMs) are abnormal, snarled tangles of blood vessels that cause multiple irregular connections between the arteries and veins. These malformations most often occur in the spinal cord and in any part of the brain or on its surface, but can develop elsewhere in the body [1]. Stereotactic radiosurgery for treatment of intracranial AVMs has been a well-established alternative to open AVM resection or embolization [2–5]. Radiosurgery uses a vascular injury response that is ideally limited to the anomalous shunting blood vessels that form the AVM nidus. The goal is to obliterate the intracranial AVM with minimal injury to the surrounding normal brain tissue [6]. For that reason, an accurate localization of the AVM on the treatment planning computed tomography (CT) is very important. Unfortunately, the AVM is typically not clearly visible on the native planning CT (which is required for accurate dosimetry) due to the low soft tissue contrast of that imaging modality in the area of interest. For that reason, alternative imaging modalities like CT angiography (CTA) or magnetic resonance (MR) angiography (MRA) can be acquired in order to identify the nidus of an AVM. Another imaging modality, which is able to visualize the nidus is the digital subtraction angiography (DSA). An advantage of DSA is its superior spatial resolution and dynamic demonstration. Therefore, DSA is typically performed for the diagnosis and determination of anatomic characterization of AVMs [6]. A DSA image is generated by subtracting a native x-ray image dataset from an image dataset acquired after the injection of a contrast agent. Assuming that there is no patient movement between the acquisition of these two image datasets, the subtraction of the datasets results in an image of the distribution of the contrast agent itself, the DSA image. Following this procedure, the native and the DSA image datasets are registered intrinsically. DSA imaging is available in two dimensions (2D) as well as in three dimensions (3D). In contrast to 3D imaging possibilities, the 2D images (typically frontal and sagittal image pairs) are acquired in fluoroscopic mode. In that way, the flow of the contrast agent within the vasculature is visualized dynamically which is beneficial in term of identifying the AVM.

In order to transfer the AVM contour from the 2D-DSA image to the planning CT, a registration of these two imaging modalities is required. A common procedure when treating an AVM with radiosurgery is to use an invasive head frame, which serves as a fixed coordinate system invasively attached to the patient’s skull [7–11]. Both angiography and planning CT are then performed with the head frame attached. Since that coordinate system (defined by the head frame) is subsequently available on both imaging modalities, i.e. planning CT as well as 2D-DSA, registration of the 2D-DSA with the CT is trivial. Both imaging modalities are acquired within the same coordinate system attached. However, such a frame makes the treatment invasive and is not favorable regarding patient comfort. In addition, the whole treatment (from imaging to radiosurgery) has to be performed in a single day, which is challenging in terms of organization and management of the required resources (staff as well as devices).

Different frameless approaches have been studied in the past. Lu et al. [12] proposed to implant fiducial markers into the patient’s skull in order to be able to register the DSA images with the planning CT. This approach has the disadvantage that the procedures is still invasive. Hristov et al. [13] introduced a method integrating digital rotational angiography (DRA) into the workflow in order to get the 2D-DSA image registered to the planning CT. This results in additional imaging dose (during DRA acquisition) for the patient in comparison to the frame-based method. Another approach was published by Steenbeke et al. [14]. They evaluated a 2D-3D match between 2D-DSA and 3D-CT within the software package XNav (Gorlachev G.E., Burdenko Neurosurgery institute, Moscow, Russia).

A similar approach was evaluated in this study. Looking for a non-invasive, frameless solution Varian Medical Systems is developing a new software prototype, called Brain Clinic (BC) in the following. The goal of BC is to register 2D-DSA images with the planning CT within a frameless workflow, i.e. without an invasive head frame serving as a well-defined coordinate system for both imaging modalities. This offers flexibility in terms of the prior described shortcomings of the so far available techniques.

The aim of this work was to evaluate a preliminary version of BC at our institution within a clinical environment by experienced professionals regarding the treatment of AVMs. The goal was to measure the target accuracy and reliability of this novel procedure based on phantom measurements as well as with the aid of real patient data. In addition, the ability to recognize mismatches within BC was assessed.

## Methods

### Software prototype BC

**Fig. 1:**
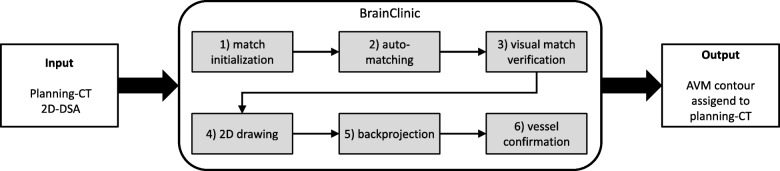
Main workflow steps in BC. The input into BC is a planning CT as wells as a set of 2D-DSA image. The output is the AVM contour assigned to the planning CT

BC is a preliminary software supporting the frameless workflow when treating AVMs. For this purpose, BC is providing tools which allow to register 2D-DSA images with the planning CT. By this, the user is able to contour the AVM on the 2D-DSA images and to transfer this contour to the planning CT. This means that the input to BC is a planning CT as wells as a set of orthogonal 2D-DSA images. The output is the AVM contour assigned to the planning CT (see Fig. 1).

The procedure within BC contains of six steps, which are described in the following:

Step 1 - Match initialization: The information visible on the 2D-DSA image is not suitable for registration with the digitally reconstructed radiograph (DRR) calculated from the planning CT, where mainly bony anatomy is visible. Therefore, the approach within BC is to register the native X-ray image (where also bony anatomy is visible) instead of the 2D-DSA image with the DRR (see step 2 below for more details). The 2D-DSA image is co-registered to the DRR afterwards by applying the resulting registration shift between the native X-ray image and the DRR to the 2D-DSA image. Having a frameless workflow in place as described in the introduction section means that the relation of the different coordinate systems corresponding to the two different images (native X-ray and DRR) is initially unknown. This circumstance is well known in radiation oncology, e.g. when registering a CT dataset with a magnetic resonance (MR) dataset. Registering these two datasets means to link the two corresponding coordinate systems of the two different imaging modalities. In case of registering two planar images acquired with a divergent beam, the zoom factor (describing the influence of the distance between the imaging source and the object on the scale of the object in the imaging plane) has to be taken into account. In our case, this means that the native X-ray image typically has a different zoom factor in comparison to the DRR. Registering the two images means that it is not enough to shift the two objects along the imaging plane but one also has to apply the mentioned zoom factor. This is exactly what the user does in the first step of the workflow using the BC prototype: Knowing the imaging directions of the two planar X-ray images out of the Dicom header, the system is generating DRRs out of the planning CT in the same directions. The user has to manually match (by translating and rotating) the native X-ray image with these DRRs (2D-2D match). Once this is done, the next step is to adjust the zoom factor of the native X-ray image such that the size of the object/patient shown on the native X-ray image is as similar as possible to the one shown on the corresponding DRR. The adjustment of the zoom factor can be done with the aid of a slider and has to be verified by the user visually.

Step 2 - Auto matching: Once the zoom factor is determined, an auto-matching algorithm is available in order to register the native X-ray image with the DRR. The algorithm in place performs a 2D-3D match, meaning that a set of DRRs is generated dynamically based on different positions and rotations of the planning CT. The best matching DRR to the X-ray image is determined afterwards and results in a translation and rotation which had to be applied to the planning CT in order to generate the corresponding DRR. These translation and rotation will be taken into account later in step 5 in order to backproject the contours on the 2D-DSA to the planning CT.

Step 3 - Visual match verification: The result of the auto-matching has to be verified by the user in the visual match verification step. In order to do so, BC is providing different tools like split/moving window tools or the ability to blend the two images.

Step 4 - 2D drawing: The approval of the registration by the user enables the 2D drawing step. The goal is to locate and contour the AVM on the frontal as well as on the sagittal 2D-DSA.

Step 5 - Backprojection: Once the AVM is contoured on the 2D-DSA images, the contours are backprojected to the CT with the aid of the prior established registration. The result is a region of interest in form of a box contour on the CT.

**Fig. 2:**
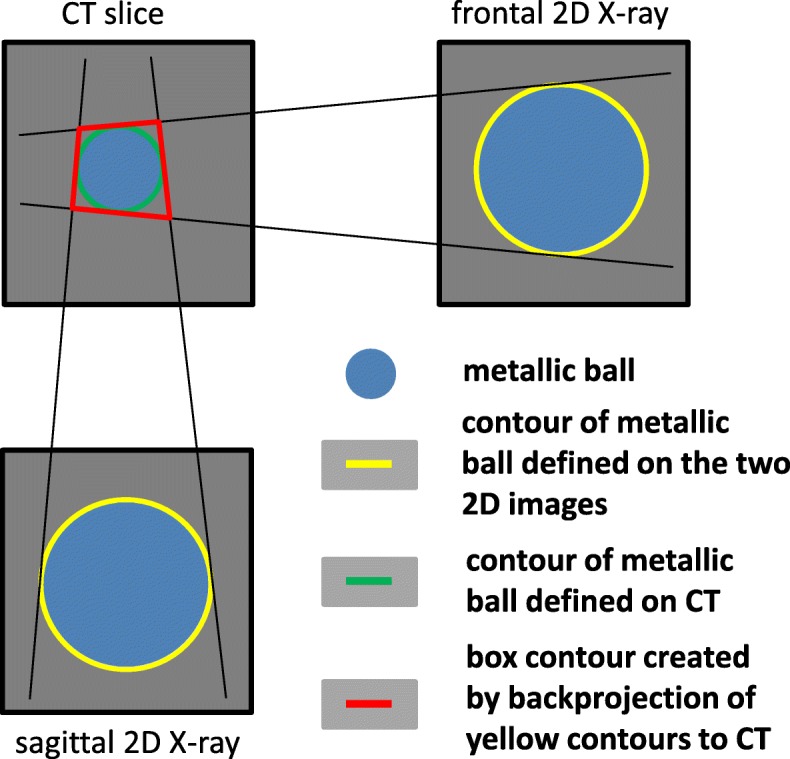
Schematic overview of the different contours used for the academic cases. The x-ray images are mimicking the 2D-DSA images in this case

Step 6 - Vessel confirmation: The box contour on the CT can be used for further refinement of the AVM contour, e.g. with the aid of additional co-registered data sets like MR images. More details are provided in section II.2. A schematic overview on how the box contour is generated is given in Fig. 2.

In order to assess the accuracy at which the box contour is determined with the aid of BC (called target accuracy in the following) and the reliability of this whole procedure a set of tests including measurements with a range from simple to rather complex test configurations were performed. These tests and measurements are described in the following sections.

### Academic case – cubic phantom

First, the workflow was tested with the aid of a simple academic case by using a cubic phantom. The cubic phantom is a homogeneous phantom containing a metallic ball of 2 mm diameter in the center of the phantom. A CT scan as well as x-ray images of the phantom were acquired. Since it was not possible to inject any contrast agent into this solid phantom, instead of the corresponding 2D-DSA images a copy of the native x-ray images were imported to BC. This is possible since the target, the metallic ball in the center, is clearly visible on the native X-ray image, mimicking an ideal 2D-DSA image. Due to the lack of matching structures, step 1 and 2 within BC were performed based on the metallic ball. Afterwards, the metallic ball in the center of the phantom was contoured on the frontal as well as the sagittal X-ray images (yellow contour in Fig. 2). These contours were backprojected to the CT resulting in a box contour on the CT. Ideally, this box contour (red contour in Fig. 2) encompasses the metallic ball on the CT. This box contour was compared with a contour drawn directly on the CT itself within the treatment planning system Eclipse (Varian Medical Systems, Inc., Palo Alto, USA) (green contour in Fig. 2), where the metallic ball is clearly visible too. A schematic overview of these contours is given in Fig. 2. The comparison of the red box contour and the green ball contour is a measure on how accurate the procedure within BC was performed. This comparison was done with the aid of an in-house analysis tool, which is described in a later section. This simple academic case reduces several error sources within the workflow to a minimum, for example errors resulting from the auto-match algorithm as well as from contouring inaccuracies.

### Academic case – anthropomorphic head phantom

**Fig. 3:**
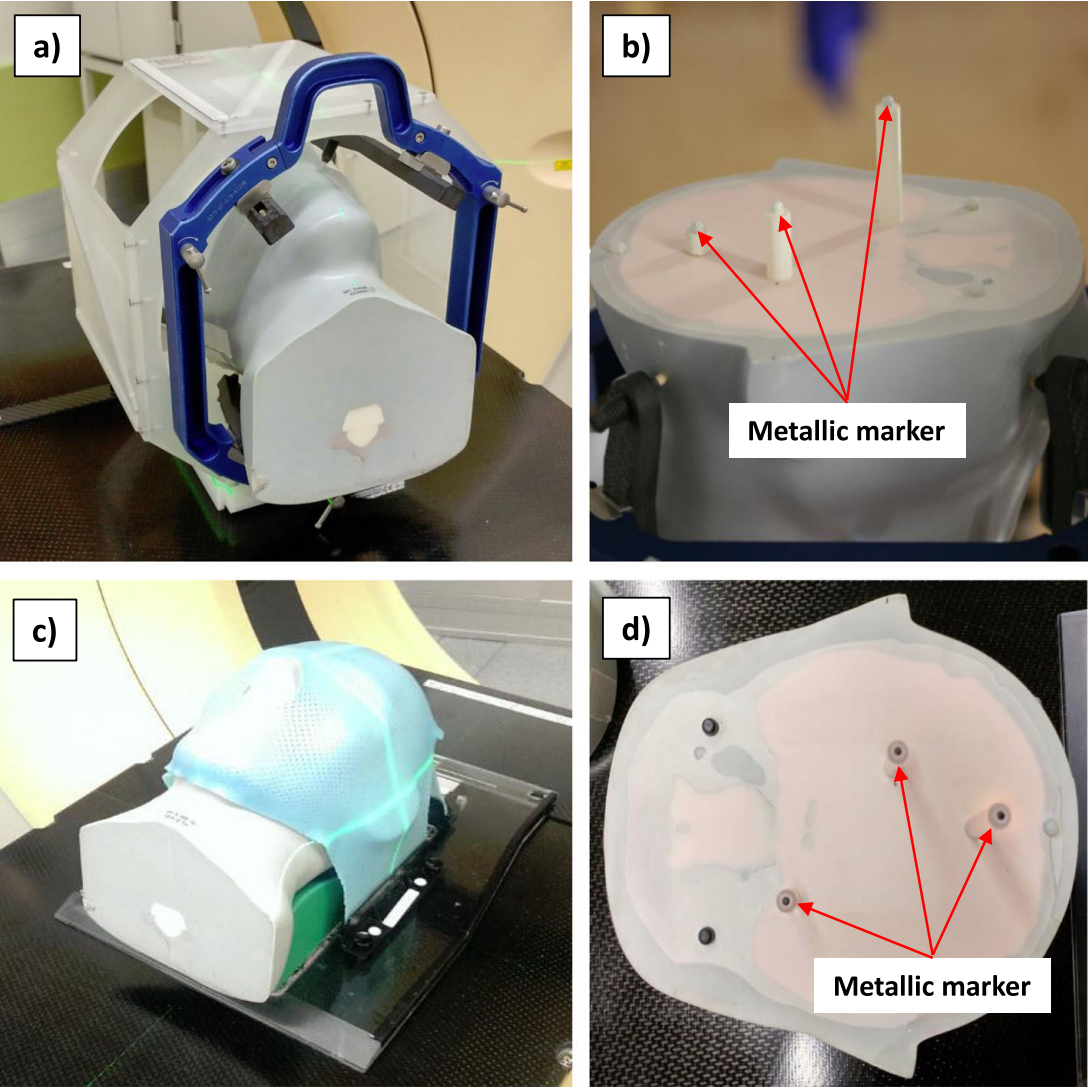
Setup of the anthropomorphic head phantom with an invasive head frame (a) and with a frameless mask system (c). Part (b) and (d) illustrate the markers and their position within the phantom

A more realistic, but still academic, situation is the case when using an anthropomorphic head phantom instead of the cubic phantom. The anthropomorphic head phantom consists of different materials to simulate bony structures and several soft tissues in the head. A cubic box can be removed and replaced by different other inserts. In this work, three metallic markers were placed inside the otherwise air filled cubic cavity. The setup of the phantom was done in two different ways: Once with the aid of an invasive head frame and once with a frameless mask system (see Fig. 3).

Similar as for the cubic phantom, a CT as well as native X-ray images were acquired for the phantom for the two different setup methods. For the invasive head frame setup only the two standard orthogonal frontal and sagittal native X-ray images were acquired during the DSA procedure. In order to see how robust BC can handle images different from this standard situation, the distance of the phantom to the imaging source, the field of view (FOV), i.e. not the whole phantom visible on the images, as well as the imaging beam directions during X-ray acquisition were varied for the frameless setup of the phantom and are described in more details in the following. One variation was to reduce the FOV. While in the standard situation, the FOV was chosen such that the whole skull of the phantom is visible on the images, the FOV is substantially reduced in this configuration. The next variation was to move the phantom laterally on the couch, which will result in different zoom factors for the sagittal images. A further variation was changing the imaging directions during DSA procedure. Once both imaging directions (frontal and sagittal) were rotated by 45° around the longitudinal axis (roll) and once by rotating both imaging directions by 30° around the lateral axis (pitch). The last variation was to rotate the phantom itself on the couch by about 30° around the vertical couch axis (yaw), while the imaging directions were not varied from standard. A summary of all these variations and the corresponding nomenclature for these setups used in this work is given in Table 1.

**Table 1:** Evaluated configurations for the anthropomorphic head phantom and the nomenclature of these settings within this report

Name	Drawing in Fig. 6	Explanation
Frame_Standard	a)	Standard patient setup with head frame.
NoFrame_Standard	b)	Standard patient setup without head frame.
NoFrame_SmallFOV	c)	As b) but with small FOV.
NoFrame_Couchshift −10 cm	d)	As b) but couch shifted laterally by −10 cm.
NoFrame_Couchshift − 5 cm	d)	As b) but couch shifted laterally by − 5 cm.
NoFrame_Couchshift 0 cm	d)	As b).
NoFrame_Couchshift 5 cm	d)	As b) but couch shifted laterally by 5 cm.
NoFrame_Couchshift 10 cm	d)	As b) but couch shifted laterally by 10 cm.
NoFrame_Roll45°	e)	As b) butimage direction rotated by 45°around longitudinal couch axis (roll).
NoFrame_Pitch30°	f)	As b) but image direction rotated by 30°around lateral couch axis (pitch).
NoFrame_Yaw30°	g)	As b) but head phantom was rotated by about 30° around vertical couch axis (yaw).

In analogy to the cubic phantom case, all workflow steps for all the different setups as described above were performed within BC. One difference in comparison to the cubic phantom was that the auto-match algorithm was used for the registration of the images in step 2, since enough structures were available for the anthropomorphic head phantom. For all setups, the three markers were contoured on the native X-ray images and were backprojected to box structures on the CT. These box contours were again compared with the contours defined in Eclipse with the aid of an in-house analysis tool (see later section).

### Patient cases

A set of 15 AVM patient cases, all of them scanned with head frame, were chosen in order to further evaluate BC under clinical conditions. Since, in contrary to the phantom cases, no clearly defined markers were available within the patients, the assessment of targeting accuracy was done by comparing the results of BC with the results of a clinically used tool, which was the treatment planning system iPlan (Brainlab AG, Feldkirchen, Germany) (reference system). iPlan is a tool which allows frame-based but no frameless workflow to integrate 2D-DSA images into the treatment planning process. Within iplan, the frame-based workflow was performed for 15 AVM cases. Within iPlan, the two orthogonal frontal and sagittal 2D-DSA images pairs are registered with the planning CT based on the invasive frame, which serves as the stereotactic coordinate system in both image data sets (2D-DSA and CT). In the same way as in BC, the region of interest is contoured afterwards on the 2D-DSA images. The backprojection process results in a box contour on the CT in the end. The imaging data of the same 15 patients are send to BC and the frameless workflow was performed as described above. By this, two box contours assigned to the same planning CT are created, one box contour generated in iPlan with the frame-based and the second box contour generated in BC with the frameless workflow. The center of masses (COMs) of the two box contours were compared.

Since often the AVM cannot be seen clearly on the 2D-DSA, there are intra-observer differences between the AVM contour drawn on the 2D-DSA in iPlan and the AVM contour drawn on the same 2D-DSA in BC. In order to take this intra-observer differences into account two different structures were contoured on each 2D-DSA (see below).

#### Control structures

**Fig. 4:**
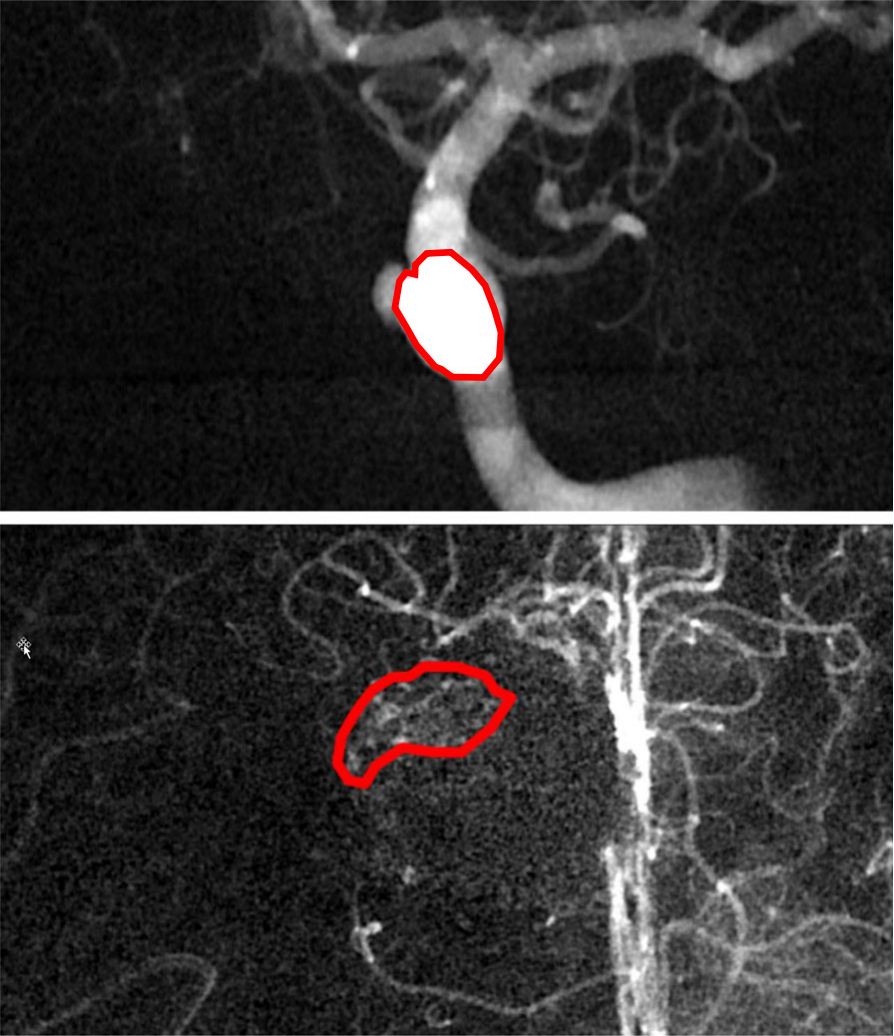
Example of a control (top) and clinical (bottom) structure for a patient case. It can be seen that it is much easier identify the control structure in comparison to the clinical structure

On the one hand, control structures were contoured on the 2D-DSA images once in iPlan and once in BC. That means that it was not the goal to contour the real AVM, but to contour a well-defined, well-visible control structure that can be contoured reproducibly in both systems. An example is shown in the upper part of Fig. 4. The control structures were contoured by a medical physicist and were backprojected afterwards to the planning CT in both systems.

#### Clinical structures

On the other hand, an experienced physician contoured all 2D-DSA images in iPlan as well as in BC. The goal was to contour the AVM in the same way as one would do it in clinical routine in this situation. In the end, consistency checks were done by visually comparing (and adapting if necessary) the contours drawn in iPlan and BC. An example of a clinical contour is shown in the lower part of Fig. 4. Both contours were backprojected afterwards to the planning CT in both systems.

### Structure comparison with the aid of an in-house analysis tool

An in-house developed software tool allows the calculation of the COM coordinates for all structures available in DICOM format. The COM coordinates were compared in each direction of the coordinate system (x, y, z) separately. In addition, the distance between the two corresponding COMs was evaluated in the 3D space (r_3D_). r_3D_ is a measure of the target accuracy of the corresponding procedure.

### Asses ability to recognize mismatches

An important error source decreasing the target accuracy comes from registration errors between CT and 2D-DSA. Since the user has to verify the registrations in step 3 in BC visually, it is important to have appropriate tools available in order to perform this verification. To check if these tools are sufficient to recognize mismatches between CT and 2D-DSA was part of this work. In order to quantify and categorize the possibility to detect mismatches (sensitivity and specificity) in the registration (2D-DSA to DRR) process statistical approaches are needed to find correlations between the observer’s answers and the level of introduced errors. To define and perform such a complete analysis would go beyond the scope of this study. Nevertheless, a tool was developed for such analysis and used for a subset of evaluations. The subset was generated as follows: Three patient cases were chosen for this evaluation part and the registration was done and evaluated within BC by an experienced physician. For this evaluation part, these three matches served as the reference registration. The reference registration was taken and the following 12 registration errors (six translational and six rotational errors) were introduced: ±0.5 mm/±0.5°, ±1.0 mm/±1.0°, and ± 2.0 mm/±2.0°, ending up in 12 defective registrations per case. For each of the three patients a set of 60 registrations was prepared out of which 24 registrations (reference registrations) were unchanged and 36 registrations had a registration error introduced. That means that the same defective match was included several times. With that, a total of 180 cases were prepared out of all three patient data sets.

In order to check if the user is able to recognize these registration errors within BC, a modified version of BC was used to evaluate this aspect. This modified version only provided a viewer, where the user can load these 180 prepared cases and where all relevant tools are available in order to evaluate these cases in terms of registration. Loading the cases was automated. A rating functionality was enabled with which the user can accept or decline the presented registration. All 180 cases were rated by three physicists and three physicians. The mismatch detection evaluation was carried out creating a database, which contains the patient case, error applied and the users rating. Using this database a filtered analysis was performed.

## Results

### Academic case – cubic phantom

The differences in the COM coordinates between the contour defined in Eclipse and the box contour received in BC was − 0.01 cm, 0.00 cm and − 0.03 cm in x, y and z direction, respectively. This results in a distance r_3D_ between the two COMs of 0.04 cm.

### Academic case – anthropomorphic head phantom

**Fig. 5:**
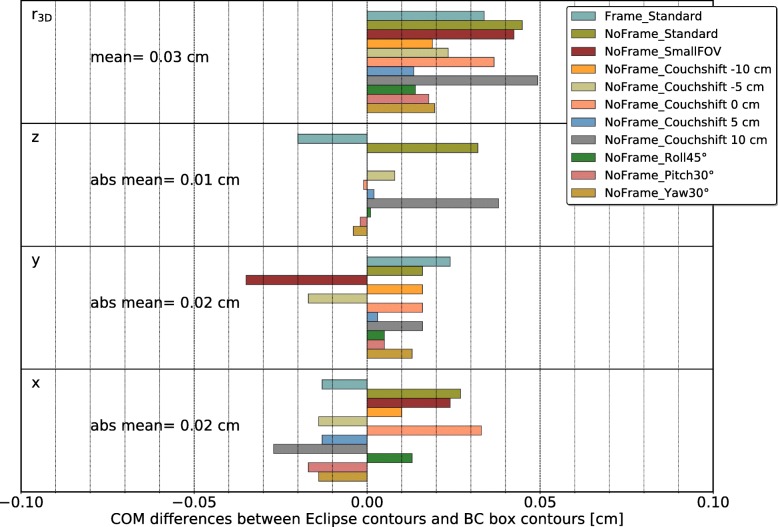
Quantitative evaluation of the anthropomorphic head phantom cases described in Table 1. The COM differences are given for all directions of the coordinate system (x, y, z) separately, as well as for the distance r_3D_

The differences between the COMs (box contour in BC and contours directly defined within Eclipse) for the 11 setups of the anthropomorphic head phantom listed in Table 1 in x, y and z direction as well as the distance r_3D_ between the COMs are shown in Fig. 5. The absolute mean values (mean of the absolute differences in x, y and z directions) over all 11 cases were 0.02 cm, 0.02 cm and 0.01 cm in x, y, and z direction, respectively. The mean distance r_3D_ over all 11 cases was 0.03 cm.

### Patient cases

#### Control structures

**Fig. 6:**
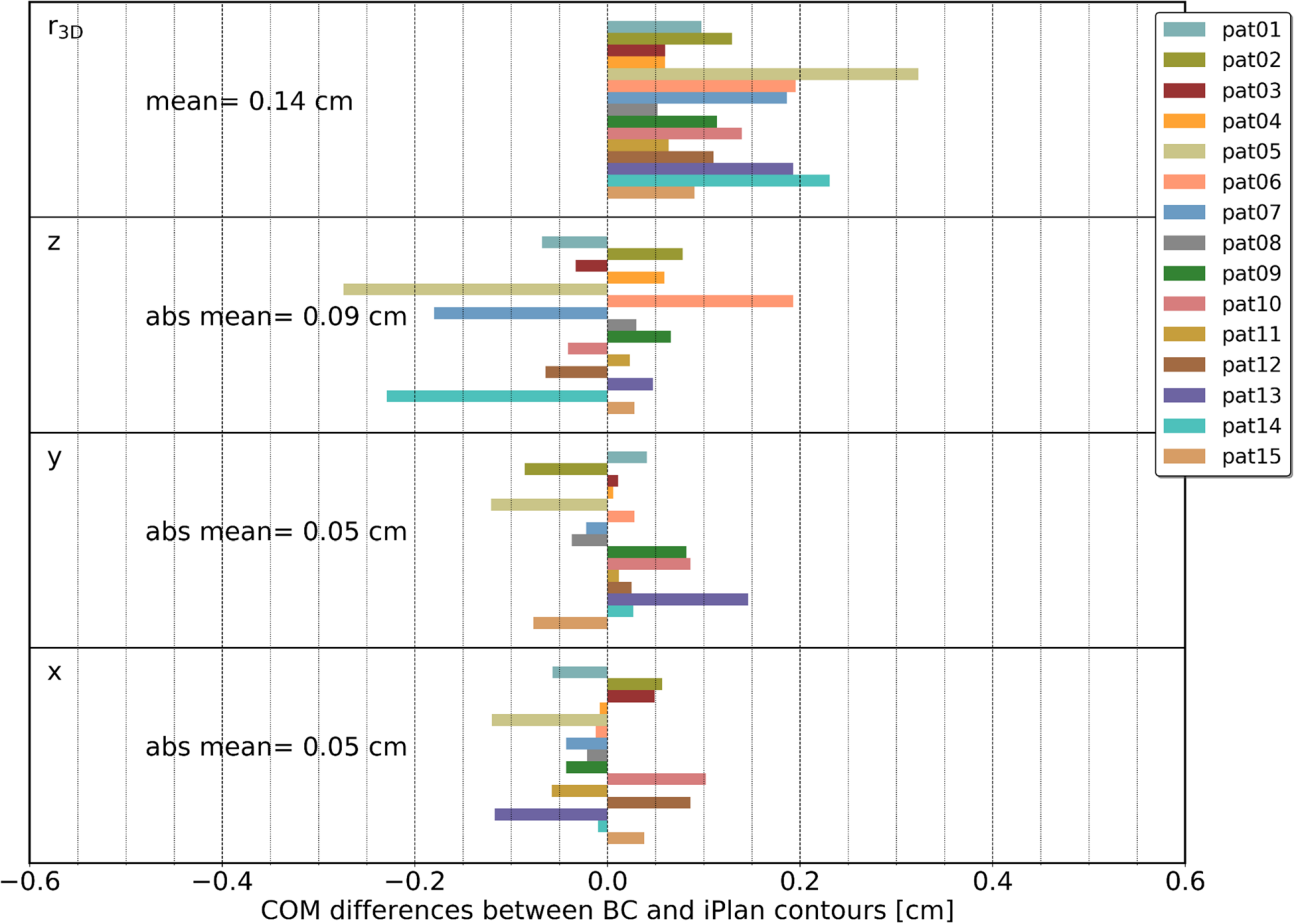
Quantitative evaluation of the 15 patient cases (control structures). The COM differences are given for all directions (x, y, z) separately, as well as for the distance r_3D_

The differences between the COMs of the control structures (box contour in BC and box contour in iPlan) for the 15 patient cases in x, y and z-direction as well as the distance r_3D_ are shown in Fig. 6. The absolute mean values (mean of the absolute differences in x, y and z directions) over all 15 patient cases were 0.05 cm, 0.05 cm and 0.09 cm in x, y, and z direction, respectively. The mean distance r_3D_ over all 15 patient cases was 0.14 cm.

#### Clinical structures

**Fig. 7:**
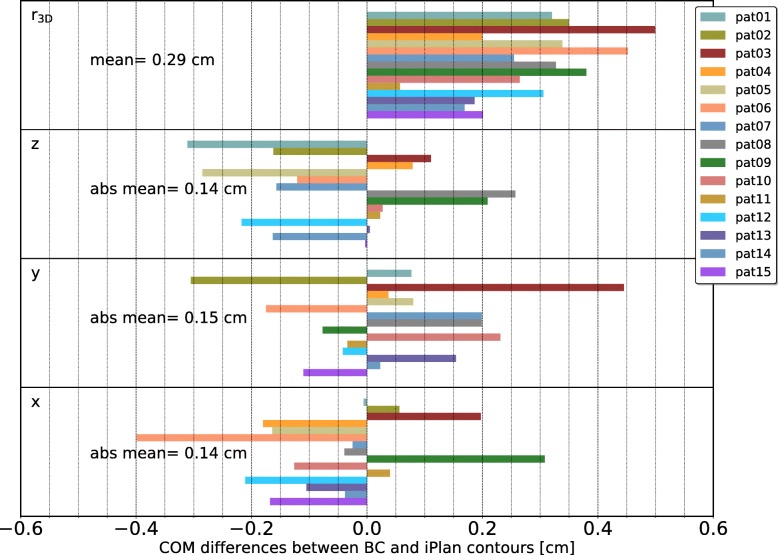
Quantitative evaluation of the 15 patient cases (clinical structures). The COM differences are given for all directions (x, y, z) separately, as well as for the distance r_3D_

The differences between the COMs of the clinical structures (box contour in BC and box contour in iPlan) for the 15 patient cases in x, y and z-direction as well as the 3D distance between the COMs are shown in Fig. 7. The absolute mean values (mean of the absolute differences in x, y and z directions) over all 15 patient cases were 0.14 cm, 0.15 cm and 0.14 cm in x, y, and z direction, respectively. The mean distance r_3D_ over all 15 patient cases was 0.29 cm.

### Asses ability to recognize mismatches

The results of the assessment of the ability to recognize registration mismatches are presented in form of histograms. The histograms show the following: The x-axis shows the magnitude of the introduced registration error (rotation or translation). The histogram itself is color-coded. The green color indicates that a registration was rated correctly by the user. The red color indicates that a registration was rated wrongly by the user. In case a user has rated all registrations correctly, all bars of the histogram would be green.

**Fig. 8:**
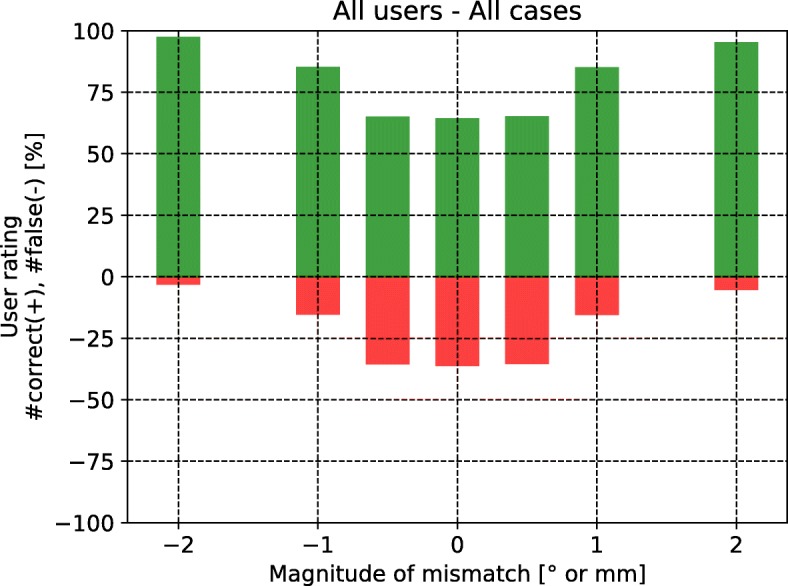
Histogram showing the rating results for all cases and for all users

Figure 8 shows the histogram of the rating results for all cases and for all users. As expected, the number of wrong rates diminishes when the magnitude of introduced error increases. About a third of the reference registrations were rated wrongly.

**Fig. 9:**
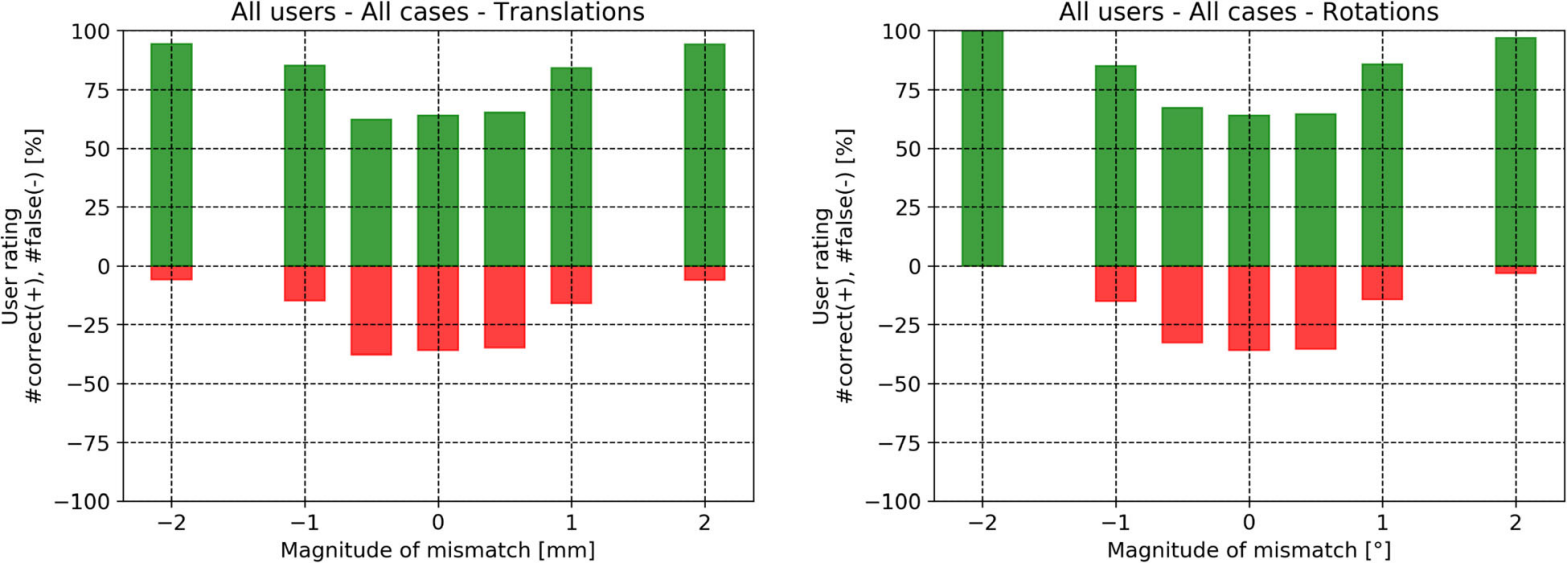
Histograms showing the rating results for all users. The cases are divided into rotations (right) and translations (left)

In Fig. 9 the histogram shows the rating results for all users where the cases are separated into rotations (left) and translations (right). The rating results look similar when comparing translations and rotations.

**Fig. 10:**
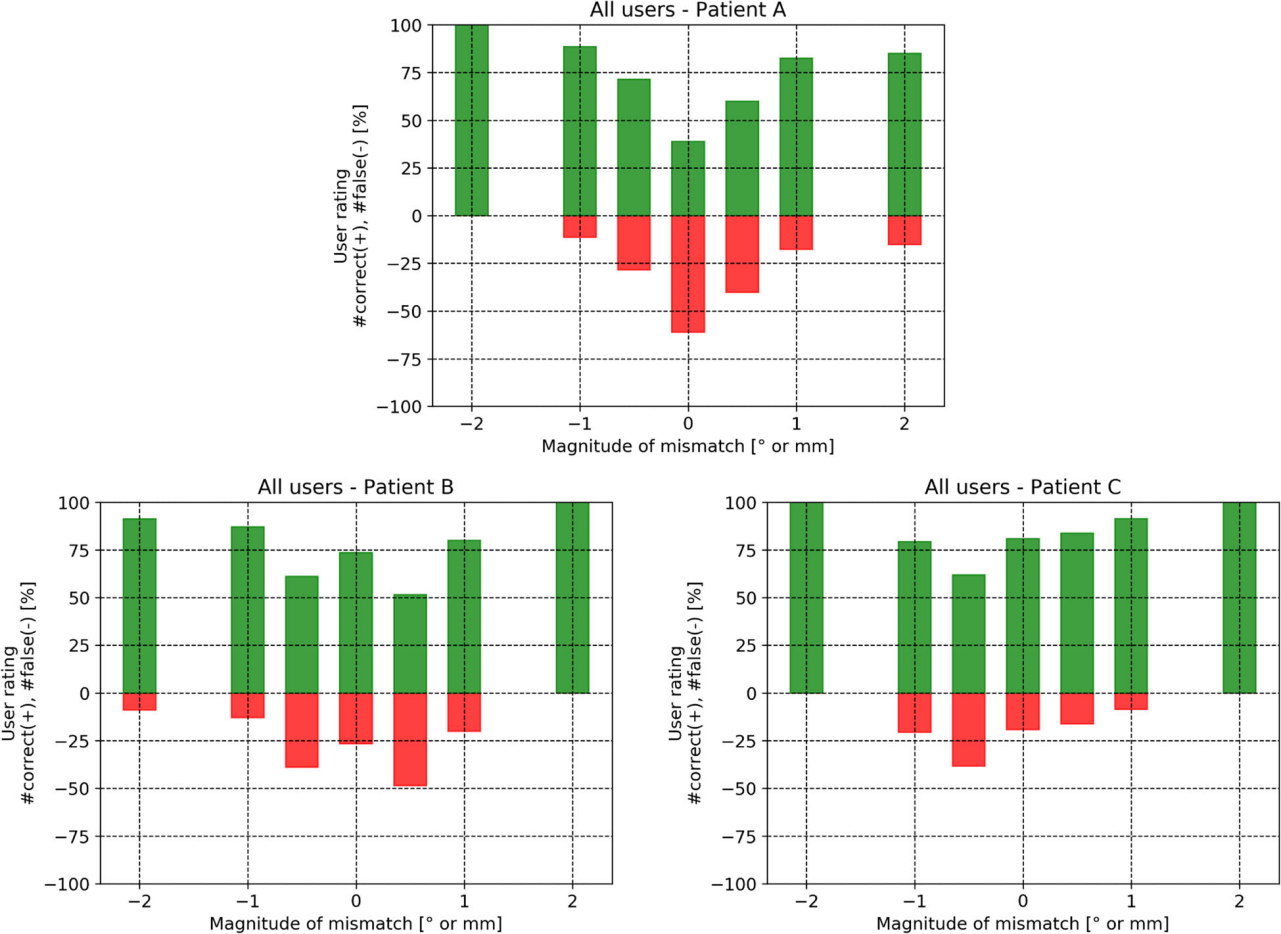
Histograms showing the rating results for all users. For each of the three patients A, B and C a separate histogram is shown

Figure 10 shows the histogram of rating results for all users, where for each of the three patients A, B and C a separate histogram is shown. About 60, 25 and 20% of the reference registrations were rated wrongly for patient A, B and C, respectively.

## Discussion

In this work, a software prototype called Brain Clinic (BC) developed by Varian Medical Systems was evaluated in a clinical environment by experienced users at Inselspital, Bern University Hospital. In order to perform this evaluation, the AVM targeting workflow was carried out within the prototype starting with simple academic cases followed by more complex, clinically relevant cases. Aim of the evaluation was to test target accuracy of the prototype and to assess the ability to recognize mismatches.

While evaluating the prototype we found that BC is a useful tool, which has the potential to fill the gap towards a frameless procedure when treating AVMs with the aid of 2D-DSA imaging in radiosurgery. The workflow is clear and straightforward.

Performing the workflow with the cubic phantom reduced the error coming from the registration to a minimum, since the academic geometry of the phantoms allows manual, very accurate and reproducible registration. The error done while adjusting the zoom factor for this phantom is larger in comparison to the errors done during the adjustments for the more realistic anthropomorphic phantom as well as for the patient data. This is due to the lack of useful structures visible on the X-ray images of the cubic phantom, which makes it hard to verify the correctness of the zoom factor visually. Nevertheless, in this simple cubic phantom errors in the adjustment of the zoom factor do have negligible impact when evaluating the center of mass of the box contour generated on the planning CT. 2D drawing and mainly backprojection (see Fig. 1) are the workflow steps remaining as prominent error sources within this academic case. The very small COM deviation between the contoured structures in Eclipse (reference) and the box contour of BC of 0.04 cm for this academic case show that these two processes, especially the process of backprojecting the 2D structures onto the CT are correctly implemented within the prototype.

Moving on to a more complex academic case (anthropomorphic head phantom) the registration step was taken into account as an additional error source within the workflow. With 0.03 cm the mean COM deviation between contoured structures in Eclipse (reference) and the box contour of BC did not increase in comparison to the cubic phantom case. For the two phantom cases, an overall mean accuracy below 0.05 cm was reached in our tests. For the anthropomorphic head phantom another goal apart from the accuracy was to check if the BC is able to handle 2D-DSA images deviating from standard settings in terms of imaging direction, field-of-view or “miss-positioning” of the phantom on the couch. For a variety of non-standard cases (see Table 1) we showed that BC is able to handle the workflow without substantially reducing the accuracy, at least for the phantom cases. The ability of BC to handle 2D-DSA images also for non-standard settings enables the possibility to use 2D-DSA images that were done for diagnostic purposes only (where the settings typically differ from standard due to better visualization of the AVM) also for radiotherapy. That means that no additional time slot for extra treatment planning images (incl. Patient preparation) would be needed anymore.

For the 15 patient cases we distinguished between contouring of control and clinical structures. Defining the AVM on the 2D-DSA images is not trivial. Contouring the AVM reproducibly in BC and in iPlan (which served as reference system in this work) is therefore difficult. The use of control structures for the comparison of BC with iPlan reduced this error source to a minimum. With a mean difference between COM for the control structures contoured in BC and in iPlan of 0.14 cm, the accuracy slightly decreased in comparison to the phantom cases. A further reduction in accuracy was observed when comparing the clinical structures due to already mentioned reasons. Mean deviation between COMs for the clinical structures contoured in BC and in iPlan was 0.29 cm.

Comparing COMs of structures of two different systems (iPlan and BC) is not straightforward. Although both systems are supporting images and structures in DICOM format, there might be small differences. That means, that the export of exactly the same structure out of the two systems may differ in slightly different dicom files and therefore introduces an error source, which is difficult to quantify. Another error source adds up when comparing COMs of the BC box structure and the iPlan box structure due to the facts, that the box structures are generated slightly different within the two systems. While the box in BC is simply the intersection of the two back-projected 2D contours, there is a post processing (smoothing) within iPlan of this box. For that reason, comparing the two box structures with the aid of e.g. dice similarity indices is not useful. However, we do not expect that this post-processing has a major influence on the COM of the structure.

The ability to recognize registration mismatches within a tool as BC is important since mismatches will potentially lead to mistreatment of the patient. In order to quantify and categorize (sensitivity and specificity) the possibility to detect mismatches, statistical approaches are needed to find correlations between the observer’s answers and the level of introduced errors. To define and perform a comprehensive analysis of this topic would go beyond the scope of this work. Nevertheless, in order to provide and test the necessary tools to perform such an analysis, we used a modified version of BC where the user is able to load and rate two already registered images (CT (DRR) with native X-ray) very efficiently. Six users of our institution rated 180 generated cases. We showed that the evaluation tool works well and that the rating procedure can be performed efficiently.

## Conclusion

While evaluating the prototype, we found that BC is a useful tool that has the potential to fill the gap towards a frameless procedure when treating AVMs with the aid of 2D-DSA images in radiosurgery. The workflow is clear and straightforward. Phantom measurements showed that the target accuracy of BC is below 0.05 cm. The patient data workflow showed that the results for BC are comparable to the results of iPlan, which is a well-established tool in clinical routine.

## Data Availability

The datasets used and/or analysed during the current study are available from the corresponding author on reasonable request.

## Abbreviations

2D: Two dimensional
3D: Three dimensional
AVM: Arteriovenous malformation
BC: Brain Clinic
CT: Computed tomography
DRA: Digital rotational angiography
DRR: Digitally reconstructed radiograph
DSA: Digital subtraction angiography
FOV: Field of view
MR: Magnetic resonance
NINDS: National Institute of Neurological Disorders and Stroke
